# Carfentanil stabilizes µ opioid receptor conformations that are ultra-efficient in inhibiting cAMP, resistant to naloxone or nalmefene but sensitive to naltrexone

**DOI:** 10.1007/s00204-025-04048-6

**Published:** 2025-05-03

**Authors:** Franziska Endt, Tao Guo, Dirk Steinritz, Niko Amend, Thomas Gudermann, Andreas Breit

**Affiliations:** 1 Walther Straub Institute of Pharmacology and Toxicology, Medical Faculty, LMU Munich, Goethestrasse 33, 80336 Munich, Germany; 2 Bundeswehr Institute of Pharmacology and Toxicology, Munich, Germany

**Keywords:** Carfentanil, Fentanyl, Morphine, CAMP, Biased agonist, Naloxone, Naltrexone

## Abstract

The highly potent opioid carfentanil (CAR) represents a growing health risk. CAR acts via G_i/o_-coupled µ opioid receptors (µOR) and exhibits ultra-high toxicity. So far, no clear association between pharmacodynamics and toxicity of CAR has been described. We created a HEK-293 cell line stably expressing the µOR and, determined ligand binding affinity (K_i_) and potency (EC_50_) of CAR, fentanyl, remifentanil, morphine or the endogenous ligand endomorphin-1. We found that µOR bind CAR with ~ 10-times higher affinity than fentanyl or remifentanil and with ~ 70-times higher affinity than morphine. Potency of CAR to inhibit cAMP was ~ 85-times higher compared to the fentanyl’s and ~ 620 higher compared to morphine. Thus, CAR’s toxicity rather associates with receptor potency than affinity. When receptor occupancy at EC_50_-values was calculated, it appeared that CAR is ~ 8-times more efficient to inhibit cAMP in comparison to morphine, fentanyl or remifentanil. Hence, we postulate that CAR stabilizes µOR conformations that are ultra-efficient in inhibiting cAMP. The OR antagonists naloxone and nalmefene are used as antidotes against opioid intoxication. Both antagonists revealed 10 to 100-times higher IC_50_-values against CAR-mediated cAMP inhibition compared to the other opioids, indicating that µOR conformations stabilized by CAR are rather resistant towards clinically used antidotes. Of note, when the long acting OR antagonist naltrexone was tested, it exhibited a ~ 65-times higher potency to inhibit CAR but not fentanyl compared to naloxone. Our data highlight the unique nature of CAR’s interactions with µOR and provide first pharmacodynamic indication that naltrexone might be a superior antidote.

## Introduction

The development of synthetic highly potent opioids such as fentanyl (FEN) has been both a blessing and a curse for the public health system. On one side, FEN’s have widely and successfully been used to ease pain or to induce sedation but, on the other side, they have also been misused as drugs due to their euphoric effects (Gustafsson et al. 2024; Khatoon and Faudzi 2024). Over time, opioid dependence dramatically increases the risk of drug overdose leading to lethal respiratory depression (Adams and Pybus 1978; Becker et al. 1976). Because FEN’s lethal dose is 50–100-times lower compared to morphine (MOR), the risk of respiratory depression after FEN intoxication is accordingly increased (Van Bever et al. 1974). The opioid receptor (OR) antagonists naloxone and nalmefene are clinically used antidotes against MOR or FEN intoxication, because, if administrated in time, they reverse the actions of an overdose until the opioid is eliminated (Drummond et al. 1977; Krieter et al. 2019; Wang et al. 1998). Interestingly, the long acting OR antagonist naltrexone, which is used to treat alcohol misuse or opioid dependence, is not in the focus for the reversal of opioid intoxications (Kirchmayer et al. 2000; Minozzi et al. 2006; O'Leary et al. 2001).

FEN differs structurally from MOR and consists of a 4-anilidopiperidine structure. Adding a carbomethoxy group to the fourth position of the FEN piperidine ring results in the formation of CAR, an ultra-potent opioid, which is 5 to10-times more toxic than FEN (George et al. 2010; Langston et al. 2020; Lust et al. 2011). In fact, CAR is so toxic that is not approved for humans but used under the brand name *Wildnil* for sedating large animals such as elephants, polar bears or rhinoceroses (Haigh et al. 1983; Zawilska et al. 2021). However, CAR has found its way on the street drug market where it is used to fortify heroin leading to thousands of additional drug deaths (Borden et al. 2023; Cowles et al. 2017; Delcher et al. 2020; Yung and Herath 2021). Additionally, it poses a serious threat to thousands of civilian lives if used as a weapon of war or terrorism (Riches et al. 2012). Even high doses of naloxone are insufficient to reverse the fatality of CAR, which makes these situations even more horrific (Leen and Juurlink 2019; Zawilska et al. 2021).

FEN and MOR act on µOR, which are G_i/o_ protein-coupled receptors expressed in neurons of the central nervous system (Pathan and Williams 2012). G_i/o_α subunits inhibit activity of adenylyl cyclase and thus reduce cytosolic cAMP levels. Those interactions between µOR and adenylyl cyclase have been proposed to inhibit the respiratory network, and thus, to contribute to the toxic actions of opioids (Manzke et al. 2003; Pattinson 2008). Further, opioids phosphorylate and thus activate extracellular-regulated kinases-1/2 (ERK-1/2) involved in cell proliferation, differentiation and analgesia (Miyatake et al. 2009; Zheng et al. 2008). G protein βγ-units released from G_i/o_α activate inwardly rectifying potassium channels and inhibit voltage-dependent calcium channels (Montandon et al. 2016). G protein-coupled receptor kinases (GRK) as well as protein kinase A (PKA) or -C (PKC) phosphorylate µOR at several serine or threonine residues, leading G protein signal desensitization, β-arrestin recruitment, receptor endocytosis and further receptor signaling (Williams et al. 2013; Zhang et al. 1996).

So far, it is not fully understood how the addition of the carbomethoxy group within the CAR molecule affects its pharmacodynamics or signaling and thus its toxicity. It has been reported that CAR binds µOR with high affinity (0.04–0.15 nM) in human brain cells or the SH-SY5Y cell line endogenously expressing OR (Costa et al. 1992; Titeler et al. 1989). In SH-SY5Y cells, an EC_50_-value of < 0.01 nM in cAMP inhibition was also observed (Costa et al. 1992). In HEK-293 cells overexpressing µOR, EC_50_-values of 0.2–8 nM were found in a BRET-based G protein activation assay, a GIRK (G protein-coupled inwardly rectifying potassium channel) activity or a β-arrestin recruitment assay (Faouzi et al. 2023; Ramos-Gonzalez et al. 2023). In line with the weak effects of naloxone after CAR intoxication, one study raised the concern that, CAR might be resistant to naloxone on the cellular level (Feasel et al. 2024).

In order to provide a rather complete analysis of CAR’s pharmacodynamics, we here established a HEK-293 cell line stably overexpressing µOR and determined ligand binding, cAMP attenuation, ERK-1/2 and receptor phosphorylation as well as β-arrestin recruitment. Further, we monitored the effects of the µOR antagonists naloxone, nalmefene and naltrexone. CAR’s actions were compared to those of FEN, MOR, remifentanil (REM) and the endogenous ligand endomorphin-1 (ENDO). When normalized to its high affinity, we found that CAR is ~ 8-times more efficient to inhibit cAMP than the other ligands, however, it was almost equally efficient in phosphorylating ERK-1/2. These data suggest that CAR stabilizes distinguishable µOR conformations, which are selectively ultra-efficient in modulating cAMP. Receptor phosphorylation mirrors receptor confirmation. We observed CAR-induced hyperphosphorylation of µOR at Thr-370 and Thr-379. Increased CAR-induced µOR phosphorylation consequently led to stronger β-arrestin-2 recruitment. When equally affine agonist concentrations were used to determine IC_50_-values for naloxone or nalmefene in cAMP accumulation, we found for both antagonists 10 to 100-fold higher values against CAR compared to the other opioids. However, compared to naloxone, naltrexone exhibited a ~ 65-times higher potency to inhibit CAR but not FEN. Thus, we report that CAR stabilizes µOR conformations that are ultra-efficient in inhibiting cAMP, highly phosphorylated, more affine to β-arrestins and resistant to naloxone and nalmefene but rather sensitive to naltrexone.

## Materials and methods

### Chemicals and antibodies

p-ERK-1/2 antibodies (Santa Cruz, Dallas, USA, E4, sc-7383), MOR (M-005), FEN (F-013), CAR (C-163), REM (R-024), ENDO (SCP-0132), naloxone (BP-048), naltrexone (PHR-8745), nalmefene (SML-2959), forskolin (FSK) (F3917) and 3-isobutyl-1-methylxanthin (IBMX) (I5879) were purchased from SigmaAldrich (St Louis, USA). [N-allyl-2,3,-^3^H]-naloxone (NET-719250UC) and [2,8-^3^H]-adenine (NET811250UC) were from PerkinElmer (Boston, USA).

### Cell culture

HEK293 cells were cultured in DMEM + GlutaMAX^™^ containing 10% fetal bovine serum and 100 U/ml penicillin and streptomycin from Gibco. In order to obtain cells stably expressing µOR a pEAK10-HA-µOR plasmid coding the rat µOR protein together with an empty pcDNA4 vector were transfected into HEK293 cells using TurboFect^™^ (R-0534) from Thermo Fisher Scientific (Waltham, USA). After 2 days, transfectants were selected with 400 µg/ml Zeocin^™^ (R-25001) from Invitrogen (Waltham, USA) for 3 weeks. Single clones were collected by using cloning cylinders, transferred to 6-well plates and resulting cell populations analyzed by detecting specific binding of [^3^H]-naloxone. µOR expression was stable over months in culture and cells proliferated by splitting them 1–10 every 72 h.

### Radioligand binding assay

At first, total membrane fractions were prepared as described previously and aliquots stored as − 80 °C (Breit et al. 2004). For saturation binding 20 µg of membranes were incubated with increasing concentrations of [^3^H]-naloxone in DMEM. Specific [^3^H]-naloxone was determined as the inhibition of total [^3^H]-naloxone by 10 µM naloxone. Samples were incubated at 37 °C for 1 h and the reaction stopped by rapid filtration over Whatman GF/C glass-fibers filters (VWR, Radnor, USA) using a cell harvester from Brandel (Gendex, Glasgow, UK). Remaining radioactivity was measured by scintillation counting using a WinSpectral1414 (PerkinElmer, Boston, USA). Competition binding assays were performed under the same conditions using 5 nM [^3^H]-naloxone as a tracer.

### Protein detection by western-blotting

Cells were seeded on poly-L-lysin (50 µg/ml) coated 6-well plates (~ 300,000/well), serum-starved after one day for 24 h and then stimulated for the indicated period of time with various opioid concentration. Cell were then placed on ice and lysed by directly adding Laemmli buffer (onefold) to the 6-well plates. Lysates were subjected to SDS-PAGE (10%) and proteins transferred to nitrocellulose (Amersham Protran^™^ 0.45 µm, #10600002, from VWR, Radnor, USA) by western-blotting. Blots were separated by a horizontal cut at 25–30 kDa. The upper part was used to detect p-ERK-1/2 by adding the p-ERK-1/2 antibody (1:500) over night at 4 °C. The lower part was used for the loading control and analysed with an anti-histone-3 antibody (1:40,000) from Abcam, Cambridge, UK (1791). After three short washing steps, blots were incubated with an anti-rabbit or anti-mouse HRP-conjugated secondary antibody (1:5,000 or 1:10,000, respectively) for 1 h at RT. After intensive washing, immune reactivity was detected by monitoring the Clarity^TM^Western ECL substrate (Bio-Rad, Hercules, USA) dependent light emission with a chemiluminescence detection system (Peqlab, Erlangen, Germany). Resulting signals were quantified densitometrically (ImageJ, RRID: SCR_003070) and ratios of ERK-1/2 and histone-3 calculated.

### β-arrestin-2 recruitment assay

 ~ 2 × 10^6^ HEK293 cells were seeded on 10 cm dishes and after 24 h co-transfected with 10 ng of a plasmid encoding a β-arrestin-2-luciferase fusion protein and 5 µg of a plasmid encoding a µOR-YFP fusion protein. After 24 h of serum-starvation, cells were detached with PBS without MgCl_2_ and CaCl_2_, resuspended in DMEM without phenolred and placed on white 96-well plates. Cells were then stimulated with 100 nM of CAR, 2 µM MOR, 10 µM of ENDO, and 1 µM of FEN or REM. After 20 min at RT, 5 µM coelenterazine h (C6780) (Thermo Fisher Scientific, Waltham, USA) was added and dual luminescence (donor emission 410 ± 60 nm, acceptor emission 515 ± 30 nm) was detected using a ClarioStar from BMG (Offenburg, Germany). BRET ratios were calculated as acceptor over donor emission. Ligand-induced β-arrestin-2 recruitment was calculated by subtracting basal values.

### µOR phosphorylation

Assays from 7 TM-Antibodies (Jena, Germany) were used in order to detect µOR phosphorylation at Ser-375 (7 TM0319 C-PA), Thr-370 (7 TM0319B-PA) or Thr-379 (7 TM0319E-PA). ~ 50,000 cells were seeded on poly-L-lysin (50 µg/ml) coated 96-well plates, serum-starved the other day and then stimulated for 5 min with the indicated opioid concentration. µOR phosphorylation was detected according to the manufacturer’s protocol.

### cAMP accumulation

*Metabolic labelling with [*^*3*^*H]-adenine (used for data shown in figures: 2 and 8)*: ~ 200,000 cells were seeded on 12-well dishes, serum-starved for 24 h prior to the experiment and labelled with 1 µCi/ml of [^3^H]adenine overnight. Cells were stimulated for 30 min at 37 °C in DMEM containing 0.5 mM IBMX, 10 µM FSK or opioids in the indicated concentration to obtain concentration–response curves and corresponding EC_50_-values for agonists. In order to obtain IC_50_-values for antagonist, equally affine opioid concentrations were co-incubated with various concentrations of the antagonist. In any case, reactions were terminated by removing the medium and adding ice-cold 5% trichloroacetic acid. [^3^H]cAMP was purified by sequential chromatography (dowex-resin/aluminium oxide columns), and detected by scintillation counting.

*alpha-screen cAMP detection kit (6760635D) from revvity (used for data shown in figure: 9)*: ~ 30,000 cells were seeded on poly-L-ysin coated 96-well plates and, serum-starved after 24 h for another 24 h. Cells were then incubated with 50 µl IBMX (500 µM) in DMEM for 5 min and stimulated with 50 µl DMEM containing FSK and IBMX, opioids or antagonists twofold concentrated. After 20 min at 37 °C, stimulation was stopped by removal of the medium and addition of 40 µl lysis puffer containing acceptor beads (12.5 µg/ml) and biotinylated cAMP (0.625 pmol). After 90 min, 10 µl lysis puffer with donor beads (12.5 µg/ml) was added and incubated for additional 30 to 60 min. Finally, acceptor bead emission (570 ± 100 nm) was detected after excitation of the donor bead (680 ± 40 nm) using a ClarioStar from BMG (Offenburg, Germany).

### Quantification and statistical analysis

Values represent the mean ± SEM of 3–6 independent experiments. Statistical analysis was performed using one- or two-sample student’s *t*-test, one‐way or two-way ANOVA followed by Tukey’s or Dunnett’s post-test using the GraphPad prism software 9.1 (RRID:SCR_002798). Shapiro–Wilk tests were performed in order to ensure normal distribution of the data sets. One symbol indicates a *p*‐value of ≤ 0.05, two of ≤ 0.01 and three of ≤ 0.001.

## Results

### CAR ultra-efficiently inhibits cAMP in HEK293-µOR cells

**Fig. 1:**
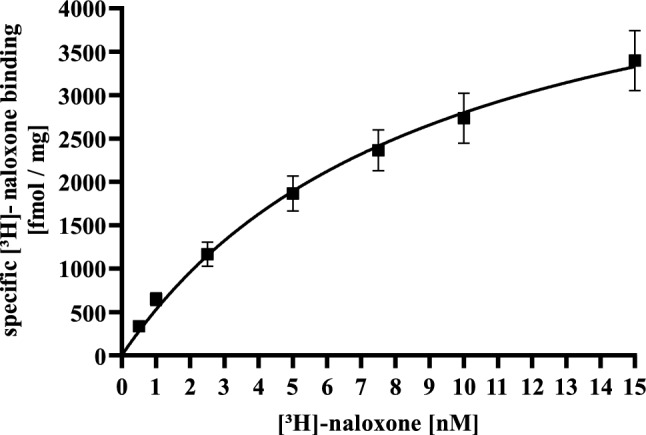
Saturation ligand binding with [^3^H]-naloxone and HEK293-µOR cells. Total membrane fractions were incubated with increasing concentrations of [^3^H]-naloxone (0.5–15 nM) with or without 10 µM naloxone for 1 h at 37 °C and specific ligand receptor binding calculated as fmol per mg total protein. Data of 3 independent experiments (N) performed in triplicates are presented as the mean ± SEM

In order to perform a detailed analysis of CAR’s pharmacodynamics under constant conditions, we first aimed at establishing a HEK293 cell line stably expressing the µOR. HEK293 cells have frequently been used to analyze pharmacodynamics of GPCRs and the µOR in general (Blake et al. 1997; Doll et al. 2011; Moller et al. 2020). Thus, after transfection of a plasmid encoding the rat µOR along with an empty pcDNA4 vector into HEK293 cells and a selection period with zeocin for 3 weeks, we obtained cell clones by single cell cloning and tested them for µOR expression by radioligand binding assay. We then chose one clone (HEK293-µOR cells) and performed saturation binding experiments with [^3^H]-naloxone. As shown in Fig. 1A, [^3^H]-naloxone binding saturated at 5.4 ± 1.1 pmol/mg, indicating robust µOR overexpression ~ 10–15-times higher compared to endogenous cell systems (Li et al. 2012; Rothe et al. 2012). Despite the high number of µOR, affinity of [^3^H]-naloxone indicated by a K_D_ of 9.8 ± 1.8 nM was very similar to previous findings obtained in endogenous cell systems (Rothe et al. 2012).

**Fig. 2:**
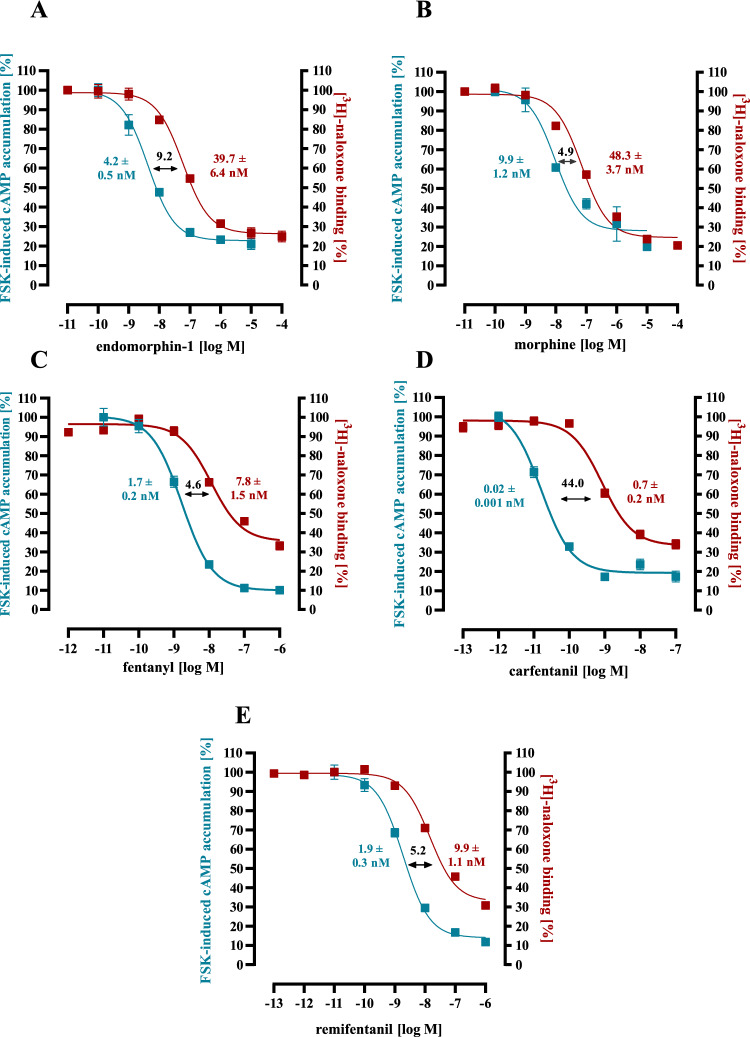
Competition binding with [^3^H]-naloxone and cAMP assay data in HEK293-µOR cells. In **a** data obtained with ENDO, in **b** with MOR, in **c** with FEN, in **d** with CAR and **e** with REM are shown. Opioid-induced inhibition (EC_50_-values) of FSK (10 µM) induced cAMP accumulation is shown in blue on the left y-axes. Competition binding (K_i_-values) with 5 nM [^3^H]-naloxone is shown in red on the right y-axes. Data of 3 independent experiments (N) performed in triplicates are presented as the mean ± SEM. Numbers between the curves indicate K_i_- over EC_50_ ratios (colour figure online)

Next, we performed competition binding experiments with [^3^H]-naloxone as the tracer and CAR, FEN, REM, MOR or ENDO as competitors. Based on these competition binding curves, K_i_-values of the opioids could be determined indicating their affinity to the µOR (Fig. 2A–E; Table 1). CAR exhibited the highest affinity with a K_i_ of 0.71 ± 0.16 nM, followed by FEN, REM, ENDO and MOR. In comparison, CAR binds µOR 10-times better than FEN and 68-times than MOR. Thus, increased affinity of CAR accounts for its higher toxicity compared to FEN but not fully compared to MOR. In order to determine potencies (EC_50_-values) of opioids to activate the µOR, their inhibitory effects on FSK-induced cAMP accumulation was analyzed (Fig. 2A–E; Table 1). CAR inhibited FSK-induced cAMP accumulation with the highest potency of 0.016 ± 0.001 nM, which was ~ 620-times higher compared to MOR. Hence, differences in the potency to inhibit cAMP rather mimics the distinct toxicity of CAR and MOR. Overall, CAR’s potency to activate µOR was 44-times better than its affinity. Such differences are commonly attributed to a phenomenon called “receptor reserve”, indicating that an agonist induces half-maximal receptor activity before it occupies 50% of the available receptors (Ruffolo 1982). In fact, when CAR’s µOR occupancy at its EC_50_-value was calculated, it occupied only 3% of the present receptors. The rank order of the potency for the remaining opioids was FEN > REM > ENDO > MOR, indicating that potency and affinity followed the same order. However, differences between affinity and potency were much smaller for FEN (4.6-fold), MOR (4.9-fold), REM (5.2-fold) and ENDO (9.2-fold). When the corresponding fractional receptor occupancy at EC_50_-values were calculated, it appeared that FEN, ENDO and REM occupied ~ 17% of the present receptors, whereas ENDO reached its EC_50_-value at 10% of receptor occupancy (Table 1). Thus, when normalized to its affinity, CAR was ~ 5-times more efficient in activating µOR compared to the endogenous ligand and ~ 8 times more than MOR or the other FENs.

**Table 1:** Competition binding experiments with [^3^H]-naloxone, cAMP accumulation assays or western-blotting experiments with HEK293-µOR cells were performed to determine K_i_- or EC_50_-values for various opioids

Opioid	K_i_ [nM]	EC_50_ [nM]			Occupancy for EC_50_ [%] (EC_50_/(EC_50_ + K_i_)) × 100		
	[^3^H]-NLX	cAMP	pERK	pERK/cAMP	cAMP	pERK	pERK/cAMP
CAR	0.71 ± 0.16	0.016 ± 0.001	0.21 ± 0.11	13.1	2.2	23.1	10.5
FEN	7.81 ± 1.52^*^	1.70 ± 0.23^*^	6.57 ± 4.32	3.9	17.9	45.4	2.5
ENDO	39.7 ± 4.15^***^	4.17 ± 0.48^***^	5.80 ± 1.42	1.4	9.5	12.7	1.3
REMI	9.86 ± 1.11^**^	1.94 ± 0.33^*^	5.06 ± 2.43	2.6	16.4	33.5	2.0
MOR	48.3 ± 3.67^***^	9.93 ± 1.24^***^	41.0 ± 11.3^***^	4.1	17.1	45.8	2.7

Asterisks indicate significant differences to CAR (one‐way ANOVA followed by Tukey’s post-test)

### CAR induced stronger β-arrestin-2 recruitment and enhanced late but not early ERK-1/2 phosphorylation

**Fig. 3:**
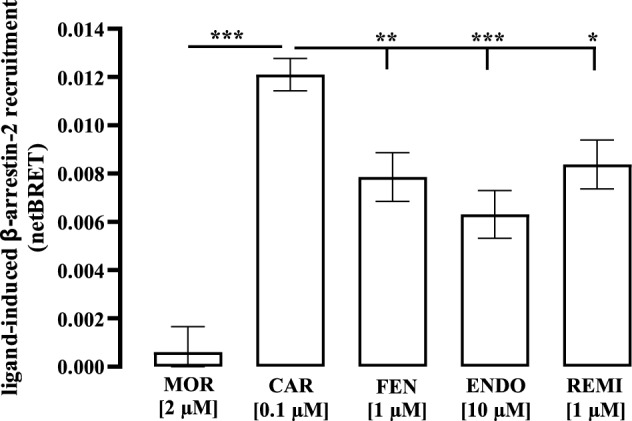
Detection of β-arrestin recruitment in HEK293 cells. Cells were transfected with β-arrestin-luciferase and µOR fusion proteins and stimulated with MOR (2 µM), CAR (100 nM), FEN (1 µM), ENDO (10 µM) or REM (1 µM). After 30 min of ligand incubation, BRET signals were detected using a ClarioStar (BMG, Labtech) after addition of coelenterazine H (5 µM). Data of 4 independent experiments (N) performed in triplicates are presented as the mean ± SEM. Statistical analysis was performed using one‐way ANOVA followed by Tukey’s post-test. Asterisks indicate significant differences to CAR

µOR-induced inhibition of adenylyl cyclase activity via Gα_i/o_ proteins has been linked to respiratory depression and thus to toxicity (Manzke et al. 2003). Mice lacking the β-arrestin-2 protein displayed reduced respiratory depression following MOR application and previous data indicated that CAR enhances β-arrestin-2 recruitment to the µOR (Ramos-Gonzalez et al. 2023). Hence, we used a BRET-based β-arrestin-2 recruitment assay and monitored agonist-dependent µOR and β-arrestin-2 interactions in HEK293-µOR cells. As expected from previous work, all opioids but MOR induced significant β-arrestin-2 recruitment after 30 min (Fig. 3) (Groer et al. 2011). In line with earlier studies, CAR promoted significantly enhanced β-arrestin-2 recruitment compared to the other opioids (Ramos-Gonzalez et al. 2023).

**Fig. 4:**
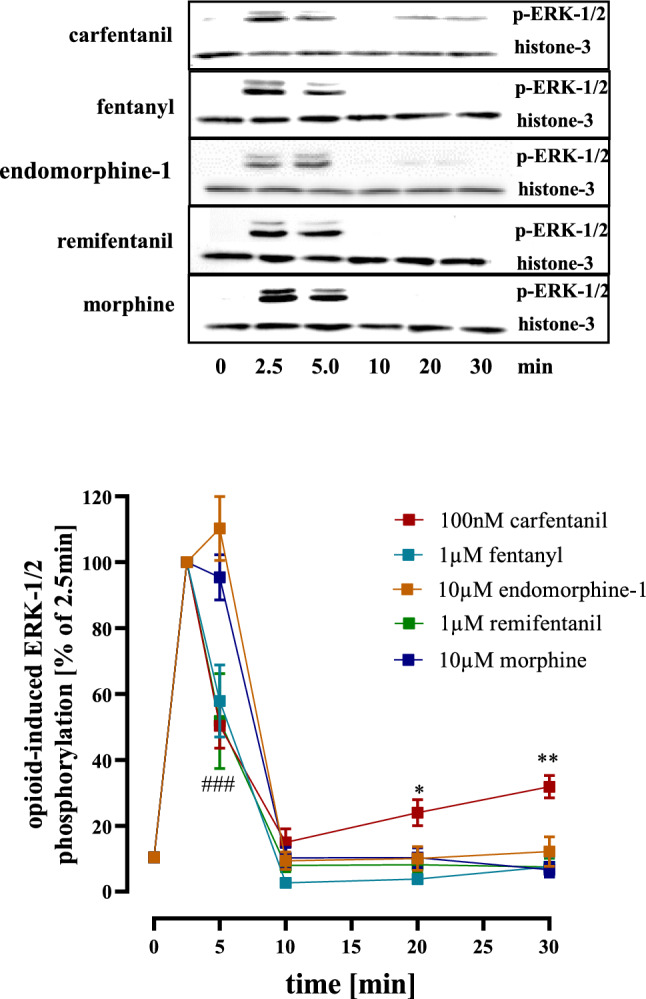
Detection of ERK-1/2 phosphorylation in HEK293-µOR cells. Upper panel, phosphorylation of ERK-1/2 was detected by western-blotting using a p-ERK-1/2 specific antibody in cells stimulated with MOR (10 µM), CAR (100 nM), FEN (1 µM), ENDO (10 µM) or REM (1 µM) for the indicated periods of times. Detection of histone-3 served as a loading control. Lower panel, after quantification with ImageJ, data of 6 independent lysates (N) were analyzed by setting p-ERK-1/2 over histone-3 ratios obtained at 2.5 min to 100%. Statistical analysis was performed using two‐way ANOVA followed by Dunnett’s post-test. Asterisks indicate in significant differences of CAR to all other opioids, hash signs indicate significant differences to CAR at time point 5 min

**Fig. 5:**
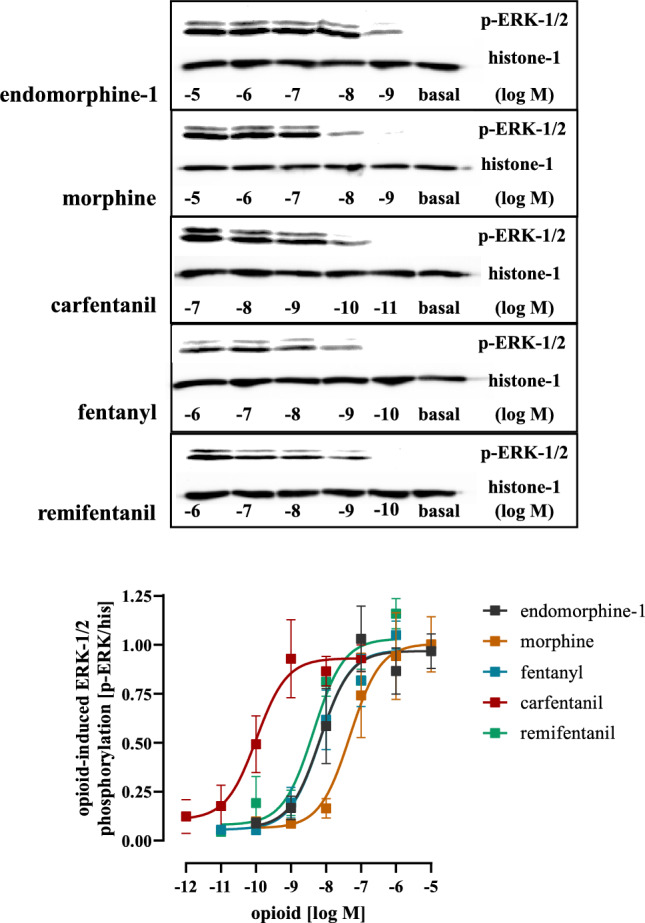
Detection of ERK-1/2 phosphorylation in HEK293-µOR cells. Upper panel, phosphorylation of ERK-1/2 was detected by western-blotting using a p-ERK-1/2 specific antibody in cells stimulated with increasing concentration of MOR, CAR, FEN, ENDO or REM for 2.5 min. Detection of histone-3 served as a loading control. Lower panel, after quantification with ImageJ, data of 6 independent lysates (N) were analyzed by calculating p-ERK-1/2 over histone-3 ratios

**Fig. 6:**
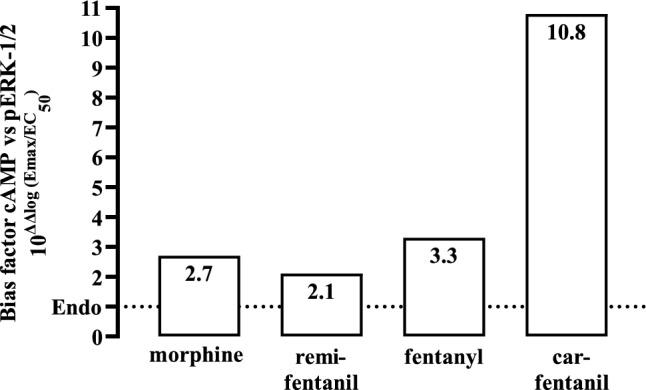
Bias agonist factors of MOR, REM, FEN and CAR were calculated using the equation and ENDO as a reference ligand: 10^log((Emax/EC50) cAMP opioid− (Emax/EC50) cAMP ENDO)) − ((Emax/EC50) p−ERK−1/2 opioid− (Emax/EC50) pERK−1/2) ENDO))^. Numbers in the bars indicate the corresponding biased factor

Both Gα_i/o_ proteins and β-arrestins contribute to opioid-induced ERK-1/2 phosphorylation (Rozenfeld and Devi 2007). In detail, Gα_i/o_ proteins induce rapid and β-arrestins delayed phosphorylation of ERK-1/2. Thus, we analyzed kinetics of opioid induced ERK-1/2 phosphorylation in HEK293-µOR cells. We observed similarly strong ERK-1/2 phosphorylation after 2.5 and 5 min for all opioids and for CAR a second and weaker peak after 20 to 30 min (Fig. 4). Because multiple studies attributed the second delayed peak in ERK-1/2 phosphorylation to arrestins (Macey et al. 2006; Tohgo et al. 2002; Zheng et al. 2008), our observations are in line with enhanced CAR-induced recruitment of β-arrestin-2. Next, we determined potencies of opioids to phosphorylate ERK-1/2 via G proteins after 2.5 min (Fig. 5; Table 1). We found CAR to be again the most potent opioid with an EC_50_-value of 0.21 ± 0.11 nM. Potency of FEN, REM and ENDO were rather similar whereas MOR had the highest EC_50_-value of 41.0 ± 11.3 nM. When potency was normalized to affinity by calculating the occupancy at EC_50_-value, we found that ENDO was the most efficient opioid to induce phosphorylation of ERK-1/2, because it required only 13% of the expressed receptors. CAR followed with an occupancy of 23%, then the remaining opioids with values of ~ 40%. In continuation, we compared receptor efficiencies of the opioids to inhibit cAMP levels and to phosphorylate ERK-1/2 after 2.5 min. We found that ENDO occupied 10% of µOR to exert half-maximal cAMP inhibition and 13% to phosphorylate ERK-1/2, indicating that µOR conformations stabilized by the endogenous ligand are rather equally efficient in activating both pathways. FEN, REM and MOR occupied ~ twice more µOR to induce half-maximal ERK-1/2 phosphorylation, suggesting a slight preference for the cAMP pathway. Of note, this factor was 10.5 for CAR, indicating that µOR conformation stabilized by CAR are much more prone to inhibit cAMP than to phosphorylate ERK-1/2. Such selectivity of an agonist to direct a given signaling pathway via its receptor is often referred to as biased-agonism and quantified by the bias factor (Kolb et al. 2022; Ramos-Gonzalez et al. 2023). Thus, we used ENDO as the reference ligand and calculated bias factors (cAMP inhibition over ERK-1/2 phosphorylation) for the FEN’s and MOR. REM, FEN and MOR showed a rather weak bias towards cAMP with factors between 2 and 3 (Fig. 6). CAR exhibited a bias factor of 10.8, further indicating that µOR conformations stabilized by CAR are ultra-efficient selectively for the cAMP but not the ERK-1/2 pathway.

### CAR induced enhanced µOR phosphorylation at Thr-370 and Thr-379

**Fig. 7:**
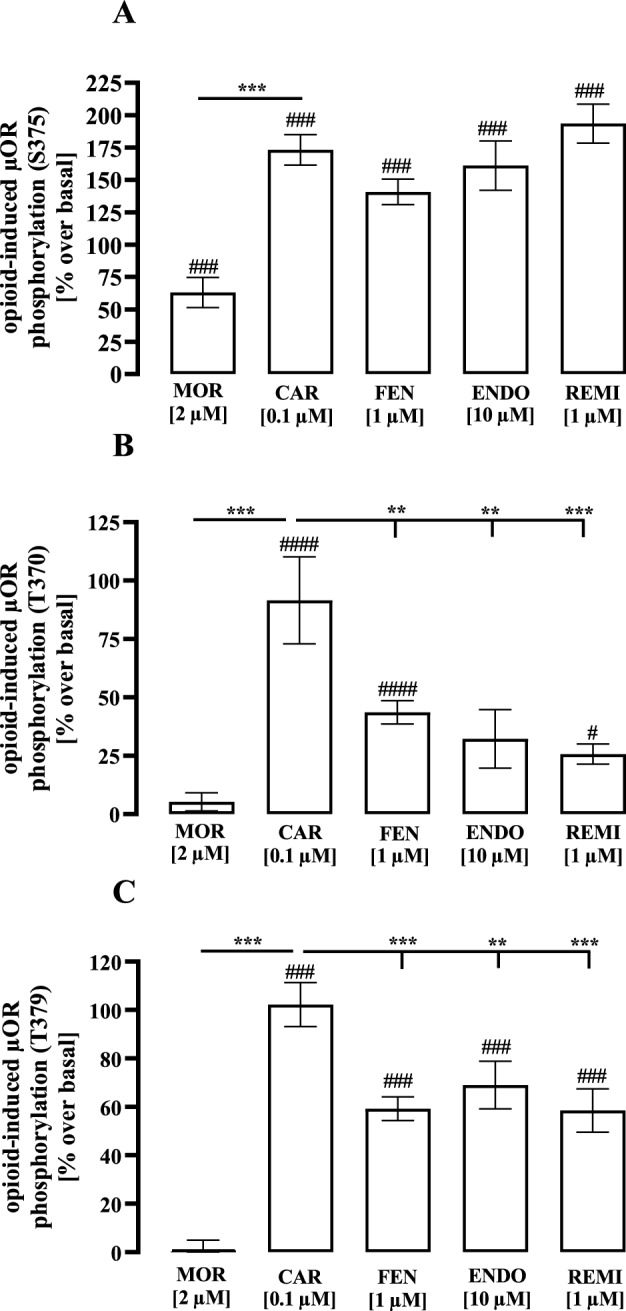
Ligand-induced µOR phosphorylation in HEK293-µOR cells. µOR phosphorylation at serine-375 (**a**), threonine-370 (**b**) or threonine-379 (**c**) were detected after stimulation of the cells with MOR (20 µM), CAR (100 nM), FEN (1 µM), ENDO (10 µM) or REM (1 µM) for 5 min using phosphorylation assays from 7 TM-antibodies (Jena, Germany). Data of 4 independent experiments (N) performed in triplicates were calculated as % over basal and are presented as the mean ± SEM. Statistical analysis was performed using one‐way ANOVA followed by Dunnett’s post-test or one-sample t-test. Asterisks indicate significant differences to CAR and hash signs to zero

Agonist exposure to µOR leads to phosphorylation of the receptor protein at various serine or threonine residues such as Ser-375, Thr-370 and Thr-379 (Fritzwanker et al. 2021; Harada et al. 1989; Pei et al. 1995). As a result, G protein activation is desensitized and β-arrestin recruitment initiated. Responsible for µOR phosphorylation are second messenger dependent kinases such as PKA and PKC and specific GRK. In particular GRK-mediated receptor phosphorylation has been shown to be agonist and receptor conformation dependent (Kelly et al. 2008). Thus, we aimed at analyzing opioid-promoted µOR phosphorylation via ELISA using phosphospecific antibodies and magnetic force-based receptor isolation. For Ser-375 robust phosphorylation was detectable for all opioids, only MOR exhibited weaker phosphorylation (Fig. 7A), which is in line with previous studies (Chu et al. 2008; Schulz et al. 2004). For Thr-370 or Thr-379, no MOR-induced receptor phosphorylation at all was detectable (Doll et al. 2011). All the other opioids induced significant phosphorylation at both threonine residues (Fig. 7B, C). Of note at both sites, CAR-induced µOR phosphorylation was significantly higher compared to FEN, REM or ENDO. These data are in line with increased β-arrestin-2 recruitment by CAR shown in Fig. 3, and provide further indications that CAR stabilizes µOR conformations that are distinguishable from those stabilized by other opioids.

### Naloxone and nalmefene exhibit reduced potency to block CAR-induced inhibition of cAMP accumulation

So far, we provided data indicating that CAR stabilizes µOR conformations that are ultra-efficient in inhibiting cAMP, highly phosphorylated and more affine to β-arrestin-2. Naloxone, the most common used antidote for opioid intoxication, has been shown to be less efficient in vivo and a recent study suggested that CAR might be resistant towards naloxone on the cellular level (Feasel et al. 2024; Leen and Juurlink 2019; Zawilska et al. 2021). Hence, we aimed at analyzing the potency of naloxone to inhibit equally affine opioid concentration in the cAMP assay.

**Fig. 8:**
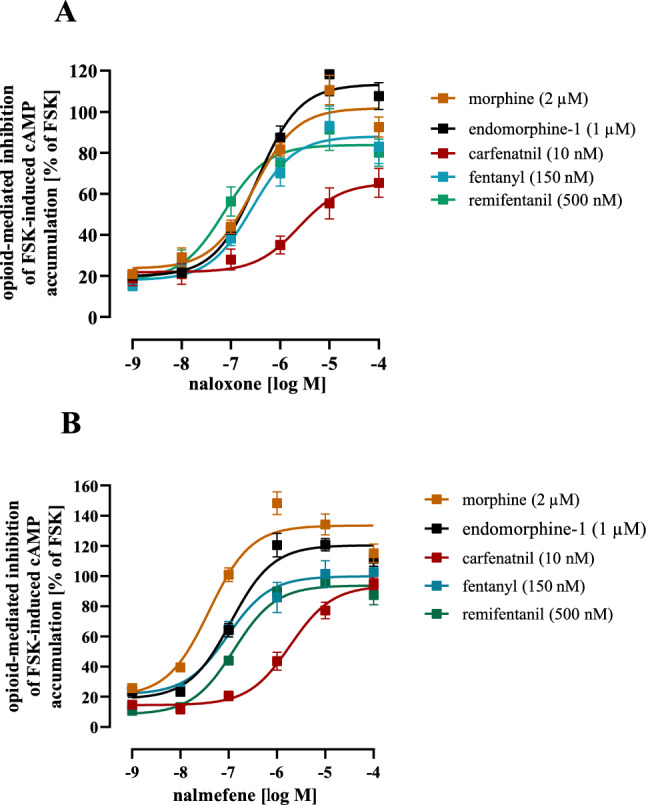
Effects of naloxone or nalmefene on opioid-induced inhibition of cytosolic cAMP in HEK293-µOR cells. MOR (20 µM), ENDO (10 µM), CAR (10 nM), FEN (150 nM) or REM (500 nM) induced inhibition of FSK (10 µM) promoted cAMP accumulation was inhibited by increasing concentration of naloxone (**a**) or nalmefene (**b**). Data of 4 independent experiments (N) performed in triplicates were compiled as % of FSK-induced cAMP accumulation in the presence of the opioid and are presented as mean ± SEM

As shown in Fig. 8A and Table 2, naloxone blocked FEN, MOR, REM and ENDO similarly with IC_50_-values between 149 ± 78 and 459 ± 244 nM. In sharp contrast, potency of the antagonist to counteract CAR was 4665 ± 1668 nM and thus significantly decreased compared to the other opioids. Interestingly, when nalmefene, a second FDA-approved µOR antagonist with slightly higher affinity was used, a very similar picture arose, with 30 to 100-times decreased potency of the antagonist against CAR compared to the other opioids. Thus, µOR conformations stabilized by CAR appeared to be rather resistant to the clinically used antidotes.

**Table 2:** Opioid-induced cAMP inhibition in HEK293-µOR cells was reversed with increasing concentrations of naloxone or nalmefene and resulting IC_50_-values of the antagonists were determined

	Naloxone		Nalmefene	
	IC_50_ [nM]	Remaining opioid effect at 100 µM	IC_50_ [nM]	Remaining opioid effect at 100 µM
MOR (2 µM)	330 ± 61.2****	− 1.8 ± 6%	33.9 ± 13.1*	− 33.4 ± 9%
ENDO (1 µM)	388 ± 57.7****	− 13.4 ± 8%	101 ± 15.8*	− 20.4 ± 6%
FEN (0.15 µM)	459 ± 244***	12.1 ± 8%	83.8 ± 14.9*	0.2 ± 4%
CAR (0.01 µM)	4664 ± 1668	34.7 ± 7%	3840 ± 1982	6.4 ± 9%
REMI (0.5 µM)	149 ± 78.1***	16.3 ± 6%	135 ± 28.2*	6.3 ± 5%

Asterisks indicate significant differences to CAR (one‐way ANOVA followed by Tukey’s post-test)

### Naltrexone inhibits CAR- but not FEN-induced inhibition of cAMP accumulation with higher potency than naloxone

**Fig. 9:**
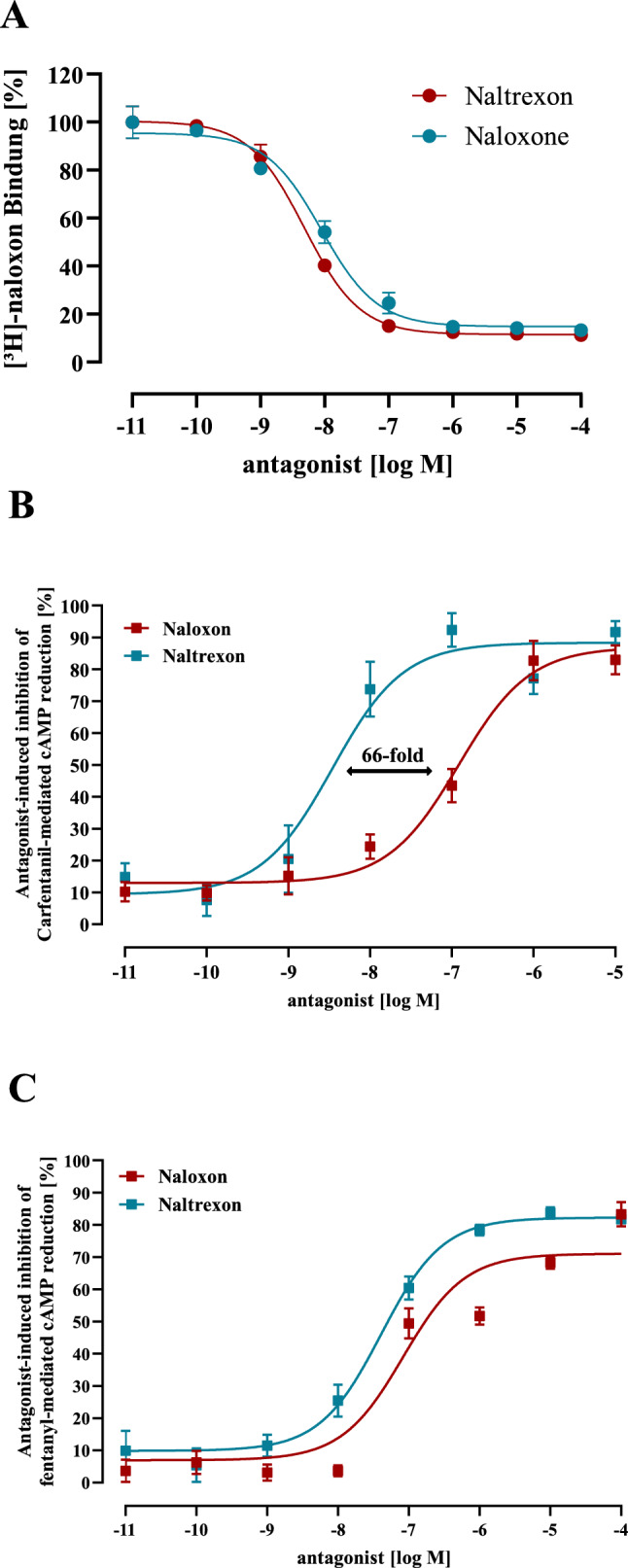
Effects of naloxone and naltrexone on ligand binding and opioid-induced inhibition of cytosolic cAMP in HEK293-µOR cells. In **a** competition binding between 10 nM [^3^H]-naloxone and increasing concentrations of naloxone or naltrexone was determined in 3 independent experiments (N) performed in triplicates. Data were normalized to % of total [^3^H]-naloxone binding and are presented as mean ± SEM. In **b** FSK-induced cAMP accumulation in the presence of 10 nM CAR of of 300 nM FEN in **c** was determined in the presence of increasing concentrations of naloxone (red) or naltrexone (blue). In **a** data of 3 and **b**, **c** of 6 experiments were compiled and presented as mean ± SEM (colour figure online)

The long acting OR antagonist naltrexone, is established as a treatment for alcohol misuse or opioid dependence (Kirchmayer et al. 2000; Minozzi et al. 2006; O'Leary et al. 2001). Its potential as an antidote for acute opioid intoxication is less explored. In order to monitor natrexone’s affinity to µOR, we first performed competition binding experiments with [^3^H]-naloxone and naltrexone or naloxone. Both antagonist showed very similar K_i_-values of ~ 3 nM, indicating similar affinities to the µOR (Fig. 9A). Based on the equal affinity, one would expect similar potency of the antagonists to inhibit CAR-induced signaling. However, when CAR-induced cAMP inhibition was reversed by increasing concentrations of either naltrexone or naloxone, naltrexone showed a 67-fold higher potency (Fig. 9B). This suggests that µOR conformations stabilized by CAR are rather sensitive to naltrexone but not to naloxone. Of note, naltrexone was twice as effective as naloxone in blocking FEN (Fig. 9C), further highlighting, that CAR stabilizes µOR confirmations distinct from those occupied by FEN.

## Discussion

Herein, we analyzed the pharmacodynamics of CAR in a HEK-293 cell line stably expressing the rat µOR in comparison to FEN, REM, MOR and ENDO. We discovered that CAR’s interactions with µOR are unique, offering new cellular level insights into the mechanisms underlying CAR’s dramatic toxicity. Further, we showed that CAR is rather resistant to the antagonists naloxone and nalmefene, and provide first data suggesting that naltrexone might serve as a superior antidote.

Fundamentally, ultra-high toxicity of CAR could be attributed to its pharmacodynamics or -kinetics. Here we focused on its pharmacodynamics and analyzed receptor affinity, potency and receptor efficiency (fractional receptor occupancy of the ligand at potency) after monitoring cytosolic cAMP levels or phosphorylation of ERK-1/2. When affinity was analyzed by radio-ligand binding experiments, we observed a rank order of CAR > FEN = REM > ENDO = MOR, with a K_i_ for CAR of 0.71 ± 0.16 nM. This value is in line with previous data observed in endogenous or recombinant expression systems (Costa et al. 1992; Lipinski et al. 2019; Titeler et al. 1989). Interestingly, CAR’s binding was only 68-times better compared to MOR, suggesting that high affinity alone cannot account for the ~ 1000-times higher toxicity of CAR. When analyzing potencies in cAMP inhibition, we found a rank order of CAR ≫ FEN = REM > ENDO > MOR, with an EC_50_-value for CAR of 0.016 ± 0.001 nM, which was 620-times lower than the one of MOR. Thus, receptor potency rather than affinity reflects the ultra-high toxicity of CAR.

A given G protein-coupled receptor such as the µOR is a bifunctional unit, that provides a ligand-binding pocket extracellularly and intracellularly a binding-site for G proteins (Kobilka 2007). Different affinities of opioids to the µOR are best explained by distinct binding modes of the opioids in the ligand-binding pocket, leading to weaker or higher stability of the ligand-receptor complex (Soldner et al. 2019; Zhang et al. 2024). Once bound to the receptor each opioid should stabilize the G protein binding site similarly, so differences in affinity among ligands should be directly transferred to distinct potencies. We observed that the differences in potency (cAMP) between CAR and MOR are 10-times higher than their differences in affinity. Thus, even when MOR and CAR bind to the same number of µOR, CAR achieves a stronger inhibition of cAMP. We postulate that CAR stabilizes µOR conformations that are highly efficient in reducing cAMP levels, most probably because they offer a particularly high-affine binding site for G_i/o_ proteins. Hence, extremely high receptor efficiency of CAR-bound µOR is a so far unappreciated feature of CAR’s pharmacodynamics and might significantly contribute to its ultra-high toxicity.

We also determined EC_50_-values of opioids in ERK-1/2 phosphorylation. Here, CAR again exhibited the highest potency. However, differences between potency and affinity were similar among distinct opioids. Thus, µOR conformations stabilized by CAR are selectively ultra-efficient in inhibiting cAMP but not in phosphorylating ERK-1/2. An agonist, that directs receptor signaling towards a particular pathway, is referred to as a biased agonist (Kelly et al. 2023; Pineyro and Nagi 2021). When the bias factor of CAR for the cAMP pathway relative to ERK-1/2 phosphorylation was calculated using ENDO as the reference ligand, a remarkable factor of 10.8 was obtained, compared to factors of 2.1 to 3.3 for the other opioids (Fig. 6). Of note, biased agonistic activity of CAR has recently been described when the synthetic ligand DAMGO was used as the reference ligand and agonist-promoted arresting binding was compared to G protein activation. Here, a bias of CAR towards arrestin binding was observed (Ramos-Gonzalez et al. 2023). In line with this study, we observed increased CAR-induced receptor phosphorylation, arrestin binding and long-term ERK-1/2 phosphorylation, which is associated with arrestin recruitment (Macey et al. 2006; Tohgo et al. 2002; Zheng et al. 2008). CAR apparently exhibits a bias towards arrestins and cAMP inhibition. On the other hand, this study did not observe a bias of CAR towards G protein activation, which at first sight might contradict our data. However, Ramos-Gonzalez et al. monitored G protein activation via BRET signals, which requires the over expression of one single Gα-subunit. G protein overexpression might mask biased signaling and CAR could bias towards other Gα-subunits that were not included in the BRET assay. Further, biased agonistic activity of CAR in cAMP inhibition could be independent of G protein activation. Finally, receptor affinity of opioids was not determined, so that receptor occupancy could not be taken into account. Consequently, further studies are required to dissect the exact molecular mechanism responsible for the bias of CAR towards cAMP signaling.

Biased agonism is based on the stabilization of particular receptor conformations, which are occupied by the biased agonist but not by “regular” agonists. This raises the question, whether these conformations are accessible for antagonists. Interestingly, a recent study raised the concern, that CAR is resistant towards naloxone (Feasel et al. 2024). Herein, we used equally affine concentrations of opioids and determined the IC_50_-values for naloxone. While the “regular” agonists were inhibited with similar IC_50_-values, a ~ 10–100 times higher IC_50_-value was observed against CAR. Interestingly, a similar picture arose, when nalmefene was used. Thus, µOR conformations that are exclusively stabilized by CAR are less accessible to antagonist treatment and, as a result, more resistant to both clinically used antidotes.

Over the years multiple OR antagonists with distinct pharmacodynamics and -kinetics have been developed. After observing that unselective antagonists like naloxone and nalmefene inhibited CAR insufficiently, we tested two µOR selective antagonists CTAP and CTOP, which also did not block CAR-induced cAMP inhibition (data not shown). Finally, we turned other attention to the long-lasting, unselective OR antagonist naltrexone, which exhibits high oral bioavailability. We found similar affinity compared to naloxone, but a ~ 70-fold higher potency in inhibiting CAR-promoted cAMP inhibition. Hence, µOR conformations stabilized by CAR are apparently more accessible to naltrexone than to naloxone. Of note, both antagonists were similarily potent in blocking FEN, further highlighting that CAR stabilizes µOR conformations distinct from those bound by FEN.

For the present study, we used a recombinant µOR overexpressing cell system to obtain a fairly complete characterization of CAR’s pharmacodynamics. This raises the question of how receptor overexpression might influence our data. There is no doubt that receptor overexpression impacts parameters like receptor affinity and efficacy. In deed substantial receptor reserve observed in HEK293-µOR cells would likely not be detectable in an endogenous expression system. However, it is unlikely that receptor overexpression selectively affects affinity or receptor efficiency of one agonist but not the other. Given the comparative nature of our study, we believe it is reasonable to assume that key findings among distinct agonists found herein are transferrable to endogenous cell systems. SH-SY5Y or F-11 cell lines have been reported to endogenously express µOR but also δOR, complicating the interpretation of data (Kazmi and Mishra 1986; Rothe et al. 2012). However, Costa et al., analyzed receptor affinity and cAMP inhibition by CAR and FEN in SH-SY5Y cells (Costa et al. 1992). They found similar K_i_- and EC_50_-values for FEN but for CAR, the EC_50_ in the cAMP inhibition was more than 10 times lower its K_i_. This strongly suggesting that ultra-high receptor efficiency of CAR is also detectable in endogenous expression systems.

## Conclusion

Our study aimed at linking pharmacodynamics of CAR with its toxicity. We observed that ultra-high affinity, potency and receptor efficiency to inhibit cAMP are associated with its toxicity. However, potency and receptor efficiency are much stronger associated with CAR’s toxicity. Our data, further suggest that CAR stabilizes particular µOR conformations that are resistant towards clinically used antidotes. Based on our data obtained on the cellular levels, we suggest that naltrexone might be a superior antidote, nevertheless further studies using endogenous cell or mouse models are required to substantiate this finding.

## Data Availability

Data are available on request from AB.

## Abbreviations

BRET: Bioluminescence resonance energy transfer
cAMP: Cyclic adenosine monophosphate
CAR: Carfentanil
ENDO: Endomorphine-1
ERK-1/2: Extracellular-regulated kinases-1/2
FEN: Fentanyl
FSK: Forskolin
GRK: G protein-coupled receptor kinases
IBMX: 3-Isobutyl-1-methylxanthin
MOR: Morphine
µOR: µ Opioid receptors
PKA: Protein kinase A
PKC: Protein kinase C
REMI: Remifentanil
